# Immune Equilibrium Depends on the Interaction Between Recognition and Presentation Landscapes

**DOI:** 10.3389/fimmu.2021.706136

**Published:** 2021-07-30

**Authors:** Daniil Shevyrev, Valeriy Tereshchenko, Vladimir Kozlov

**Affiliations:** ^1^Laboratory of Clinical Immunopathology, Research Institute for Fundamental and Clinical Immunology, Novosibirsk, Russia; ^2^Laboratory of Molecular Immunology, Research Institute for Fundamental and Clinical Immunology, Novosibirsk, Russia

**Keywords:** adaptive immunity, immune equilibrium, T-cell receptor repertoire, B-cell receptor repertoire, antigen presentation/recognition, homeostatic proliferation, a rank-size frequency distribution of T- and B-cell receptors, immunopeptidome

## Abstract

In this review, we described the structure and organization of antigen-recognizing repertoires of B and T cells from the standpoint of modern immunology. We summarized the latest advances in bioinformatics analysis of sequencing data from T and B cell repertoires and also presented contemporary ideas about the mechanisms of clonal diversity formation at different stages of organism development. At the same time, we focused on the importance of the allelic variants of the HLA genes and spectra of presented antigens for the formation of T-cell receptors (TCR) landscapes. The main idea of this review is that immune equilibrium and proper functioning of immunity are highly dependent on the interaction between the recognition and the presentation landscapes of antigens. Certain changes in these landscapes can occur during life, which can affect the protective function of adaptive immunity. We described some mechanisms associated with these changes, for example, the conversion of effector cells into regulatory cells and *vice versa* due to the trans-differentiation or bystander effect, changes in the clonal organization of the general TCR repertoire due to homeostatic proliferation or aging, and the background for the altered presentation of some antigens due to SNP mutations of MHC, or the alteration of the presenting antigens due to post-translational modifications. The authors suggest that such alterations can lead to an increase in the risk of the development of oncological and autoimmune diseases and influence the sensitivity of the organism to different infectious agents.

## Introduction

The immune system is a complicated multilevel system of protection from different pathogens that contributes to the multicellularity and maintenance of genetic homeostasis (1–3). The development of adaptive immunity is associated with the appearance of RAG (recombination-activating gene) and two consecutive whole-genome duplications (4) that could be associated with the appearance of vertebrates and a transition from Agnatha to gnathostomes, which occurred around 500 million years ago (5, 6). The most important evolutionary advantage of adaptive immunity seems to be its specificity, which provides high precision and selectivity of the immune system activity. Another important advantage is the formation of immunological memory, which provides a quick and targeted reaction to the pathogen that the organism faced before (7). The so-called “price” that has to be paid for these advantages is the necessity to re-customize the adaptive immunity and form the immune memory individually in each generation.

The evolutionary development of the adaptive immune response is associated with the appearance of populations of T and B lymphocytes. Their precursors are found at the stage of early vertebrates (8). The main peculiarity of adaptive immunity is the formation of T and B lymphocytes with a high diversity of clones, wherein each clone has a unique antigen-recognizing receptor (TCR—T-cell receptor or BCR—B-cell receptor, respectively). For example, according to the recent data, the clonal diversity of only *β* chains of TCRs in the peripheral bloodstream is up to 10^8^, which does not reflect the whole diversity that is comprised in the different organism compartments because the total number of T cells in the human organism is up to 10^12^ (9). At the same time, a recent evaluation indicates that the potential diversity of *αβ* TCRs varies from 10^20^ to 10^61^, which significantly exceeds the number of unique TCRs in the human organism (10, 11). In the case of B cells, the potential diversity of the BCR repertoire is also great and reaches 10^20^ (12–14). However, as for TCRs, the actual number is lower than the theoretical one and is approximately 10^8^–10^9^ of unique heavy BCR chains in the peripheral bloodstream (15, 16). These peculiarities lead to high personalization of repertoires, when the major part is private TCRs/BCRs and only a small part can be common in different individuals (public TCRs/BCRs). It is suggested that cross-reactivity plays an important role in the recognition of antigens because the approximate diversity of potential antigens reaches 20^9^ and seems to exceed the summed actual diversity of T- and B-cell repertoires (11).

Such diversity is provided due to recombination of V(D)J gene segments of TCRs and BCRs caused by the activation of the RAG gene and due to the effect of terminal deoxynucleotidyl transferase (TdT) at the early stages of lymphocyte maturation (17, 18). The migration of T cells from the thymus begins at the end of the first trimester of the intrauterine development, while TdT, which randomly inserts nucleotides during V(D)J recombination, begins to express only in the middle of the second trimester (19). Thus, the majority of T cells in the fetus to the middle of gestation have zero nucleotide insertion in the region CDR3 (complementarity-determining region-3) (20). Still, their repertoire has a quite high diversity of TCRs due to V(D)J recombination. Unlike TCRs, the diversification of the BCR repertoire occurs earlier. For this reason, at the beginning of the second trimester, the BCR repertoire is characterized by a relatively high diversity, which gradually increases to the time of birth. After the birth, the diversity of the T-cell repertoire continues to increase to the involution of the thymus, while an increase in the diversity of the B-cell repertoire seems to be limited by the age-related degeneration of the bone marrow (20, 21). At the same time, at the early stages of development (less than 14 weeks), both repertoires of T and B cells are characterized by the oligoclonal organization that is replaced with polyclonal one by the 17th week of gestation, which is associated with a progressive increase in the number of sjTRECs (signal-joint T-cell receptor excision circles) and sjKRECs (signal-joint kappa-deleting recombination excision circles) (20). In early childhood, the diversity of T- and B-cell repertoires tends to its maximum (20, 22). Thus, in the fetal period and early childhood, the main diversity of the TCR and BCR repertoires is established that form the general landscape of recognition of antigens, which normally changes insignificantly within the life and tends to decrease with aging (22–24).

It is worth noting that MHC (major histocompatibility complex) molecules influence the formation of TCR repertoires of CD4^+^ and CD8^+^ lymphocytes. In other words, allele variants of MHCs limit the diversity of the represented antigens, which in turn, influences the formation of naïve and antigen-experienced TCR repertoires (25–27). At the same time, there are some differences in the formation of TCR repertoires of CD4^+^ and CD8^+^. This could be associated with different events observed in the thymus that determine the choice between the CD4^+^ or CD8^+^ cell differentiation. Double-positive CD4^+^CD8^+^ lymphocytes that receive a strong TCR-MHC-II signal, quickly stop the expression of CD8 and become single-positive CD4^+^ lymphocytes. In turn, CD4^+^CD8^+^ lymphocytes that do not receive a relatively strong TCR-MHC-II signal for a long time stop the expression of CD4^+^ and become single-positive CD8^+^ lymphocytes (28). Thus, CD8^+^ lymphocytes undergo a stricter selection in the thymus. Along with the possibility of recognizing epitopes presented by MHC-I, they lose the capability to recognize epitopes presented by MHC-II, which plays an important role in forming the naïve CD8^+^ TCR repertoire (29). This is confirmed by a small total amount of common TCR*β* sequences in the populations of CD4^+^ and CD8^+^ lymphocytes, *i.e.* TCR repertoires of CD4^+^ and CD8^+^ overlap weakly, and there is a small amount of TCRs capable of reacting with both classes of MHC-I and MHC-II (30). Thus, the specificity of TCRs at the stage of CD4^+^CD8^+^ cells regulates the choice of CD4/CD8 differentiation. Further changes in the CD8^+^ repertoires could be associated with the allele variants of MHC-I, in particular, with their variants of fastidious or promiscuous binding that initiate oligoclonal or polyclonal variants of the immune response, respectively, by changing the number of certain clones (31–33). The difference between the CD4^+^ and CD8^+^ repertoires is in the formation of T-regulatory cells (Tregs) with a relatively high affinity of TCRs to self-antigens at the double-positive stage (34). Thus, the CD4^+^ repertoire contains cells with relatively high affinity to self-antigens, which is not observed in the CD8^+^ repertoire.

The formation of the naïve BCR repertoire of B cells is not so dependent on innate immunity. Similar to T cells, during maturation, B cells go through several stages of positive and negative selection. Each B cell can go through several cycles of rearrangement of V genes at different stages of maturation to increase the possibility of the formation of BCRs with the minimal capacity of reacting to self-antigens for the population of B-2 cells and a relatively higher affinity to self-antigens for the population of B-1a and B-1b cells (35, 36). Further formation of antigen-experienced BCR repertoire occurs in the process of somatic hypermutations (SHMs) during the maturation and activation of B cells on the periphery and under the mediated effect of Th cells, which suggests indirect involvement of the innate immunity (36).

Although the formation of the diversity of Ag-recognizing receptors occurs in a stochastic manner due to random V(D)J recombination and non-template nucleotide insertions (NIs), it is limited by a set of allele variants of MHC genes for a certain subject, at least, for CD4^+^ and CD8^+^ cells. Probably, this determines the individuality of the immune response and peculiarity of the homeostasis of the immune system in general in a certain organism. In this review, the authors will shortly describe the main peculiarities that can occur throughout life and affect the immune equilibrium increasing the risk of pathology.

## Landscape of Recognition

As it was mentioned before, the general landscape of recognition is understood as a diversity of specific antigen-recognizing receptors that include TCRs of CD4^+^ and CD8^+^ cells, as well as BCRs of B cells. It is assumed that the higher the diversity of antigen-recognizing receptors, the wider the range of antigens that can potentially be recognized by the immune system, and, thus, the higher the effectiveness of the immune response against pathogens and altered self-antigens (37). It is suggested that the maintenance of auto-tolerance and the efficiency of anti-cancer immunity are also associated with the diversity of antigen-recognizing receptors of Tregs, which is critical in the context of antigen-specific action of these cells (38). Since the number of cells in the organism is limited, the formation of protective diversity should be based on the relation between the general diversity and the size of each antigen-specific clone. This idea is described in the Protecton Theory (39), wherein the protecton is the minimum number of cells of certain antigen specificity required for the timely provision of the sufficient number of effector cells per the unit of the body volume for the efficient protection against antigens (10, 39). Thus, knowledge of the clonal organization of T- and B-cell repertoires is important for the understanding of consistencies in the immune response in normal and pathological conditions, as well as for the identification of the peculiarities of the immune equilibrium maintenance in different conditions.

## Organization of the Human T-Cell Repertoire

**Figure 1:**
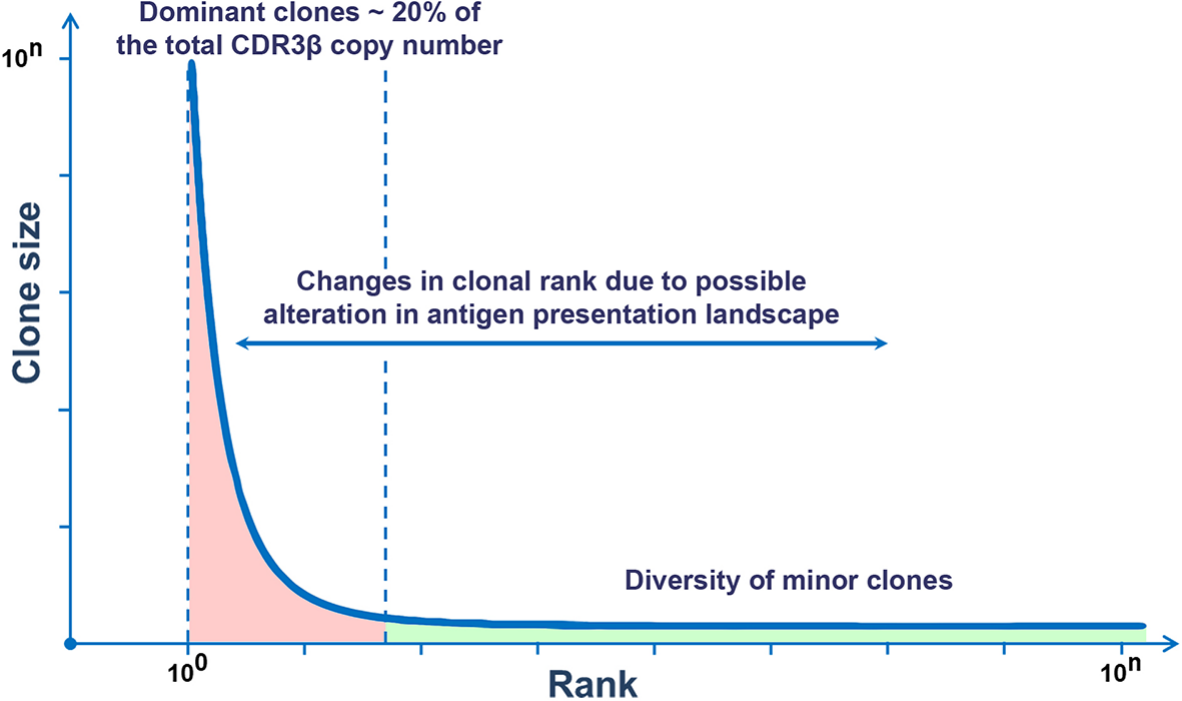
Rank–size frequency distribution follows a power-law distribution. This graph demonstrates the ranking of TCR clones by sizes. To the left, there are few dominant clones (red), and to the right, there is the long tail reflecting a multitude of minor clones (green).

Recently, next-generation sequencing technologies and mathematic analysis have expanded the understanding of the clonal organization of the TCR repertoire in humans. It has been shown that the distribution of T-cell clones in the general TCR repertoire complies with the general consistency pattern within the human population and is not age-dependent (22, 40). The distribution of clones in the descending rank order (r) depending on the size of a clone (C) is subject to power-law distribution, *i.e.* the rank (r) of the largest clones correlates with their size (C) according to the power-law distribution r~C^-α^, wherein *α* is a scaling exponent (22, 41). The size of a clone is inversely proportional to its rank, *i.e.* the larger the clone, the lower is its rank, and *vice versa*. This dependence is described by a power-law function y = kC^-α^ (40, 41). Empiric calculation of *α* is associated with a number of difficulties and depends on the used methods of sequencing and mathematical analysis. Thus, a recent study on the frequency distribution of T-cell clones of two independent cohorts showed a power-law relationship between the rank and size of the largest clones. At the same time, in both cohorts, *α* was almost similar and was equal to ~1.2 (22). The character of distribution of T-cell clones was similar in people of different ages. Thus, the general TCR repertoire in different people is characterized by a similar frequency distribution of T-cell clones regardless of age and is represented by a small number of dominant clones and a large variety of minor clones, which is consistent with the general type of Pareto distribution (**Figure 1**) (42).

A significant part of dominant clones in healthy people comprises zero insertion clones that are formed before birth and preserve in high abundance for several years with a tendency to a slight decrease throughout life (22, 24). At the same time, these clones provide the basis for public TCR repertoire in different individuals, which raises the issue of the presence of the inborn evolutionary determined set of T-cell clones within the adaptive immunity (11, 22, 24). If these clones exert certain functions or if they are a by-product of the formation of the TCR repertoire in the fetal period, it should be the subject of further studies.

**Figure 2:**
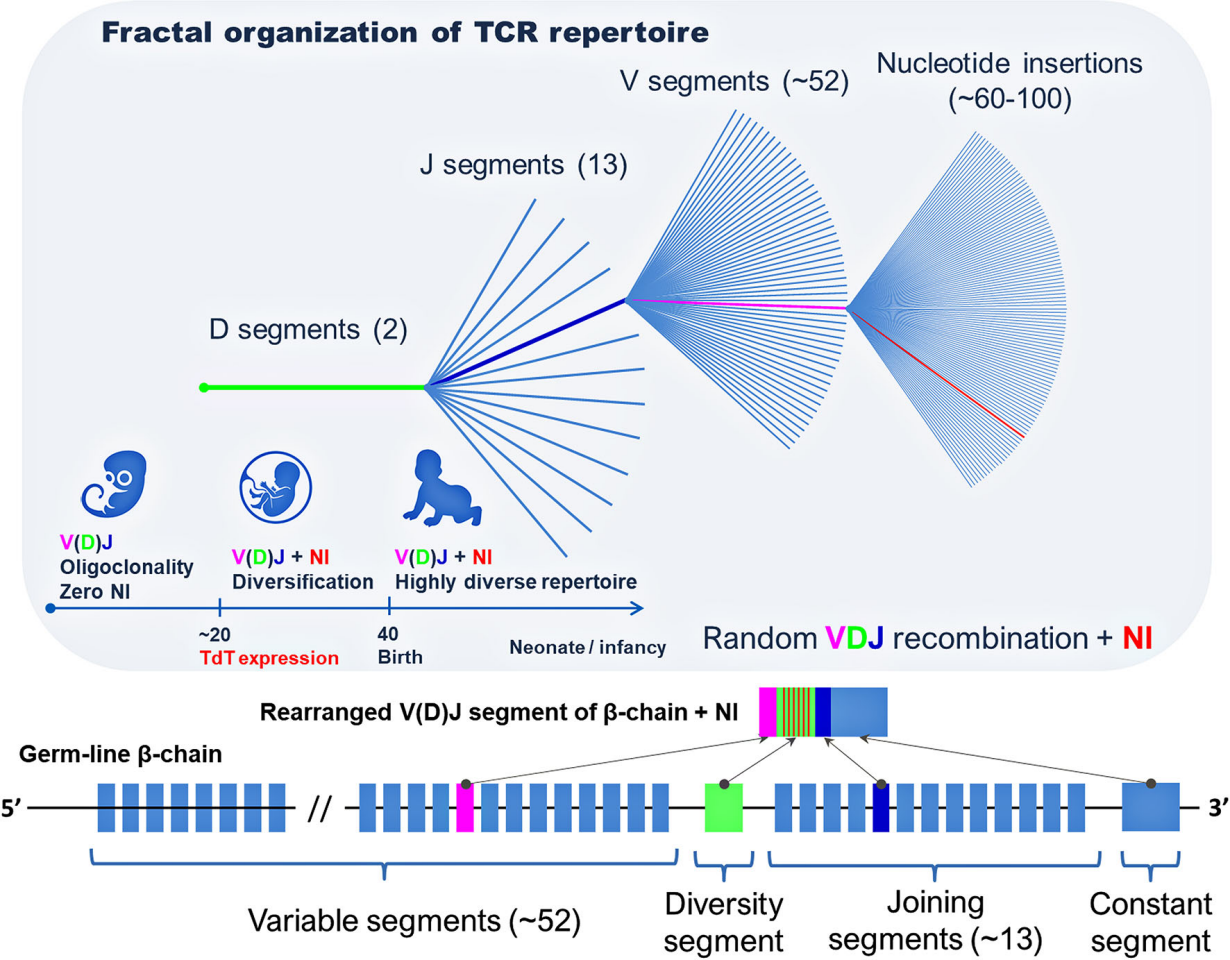
Fractal organization of T-cell repertoire. A fractal is a set with self-similarity (an object that exactly or approximately coincides with a part of itself, similar to itself on any magnification).

Meier et al. (40) studied the frequency distribution of the TCR*β* sequences at each level of the combination of gene segments (DJ, VDJ, and VDJ + NI) and revealed a fractal organization of the TCR repertoire and self-similarity of the frequency distribution of unique TCR clones (**Figure 2**) (40). An earlier study revealed the fractal organization of CD8^+^ TCR repertoires (43). At the same time, persons that are similar by human leukocyte antigens (HLAs) have a similar organization of TCR repertoires, which confirms the involvement of MHC genes in the formation of self-similarity pattern with a strict hierarchy of dominant and minor clones in the individual TCR repertoire (40).

Besides, this study showed that the development of the “graft-*versus*-host disease” (GVHD) in recipients after transplantation of hematopoietic stem cells (HSCs) is associated with the changes in the clonal organization of the TCR repertoire and change of dominant clones within the first four ranks in comparison with the respective donors (40). Probably, such a shift in dominant clones is associated with incomplete identity by MHC genes between donors and recipients. GVHD is based on the incompliance between the landscapes of self-antigen presentation, which leads to the activation and expansion of minor self-reactive clones in recipients with GVHD (40, 44). This can be associated not only with quantitative changes in the clonal organization of the TCR repertoire but also with the plasticity of some subpopulations of T cells and the respective functional changes within these subpopulations (45, 46). In particular, the transition of some Tregs to some subpopulations of the effector cells or polarization of Th0 into Th17 cells in HSC recipients can lead to the development of GVHD (46, 47).

**Figure 3:**
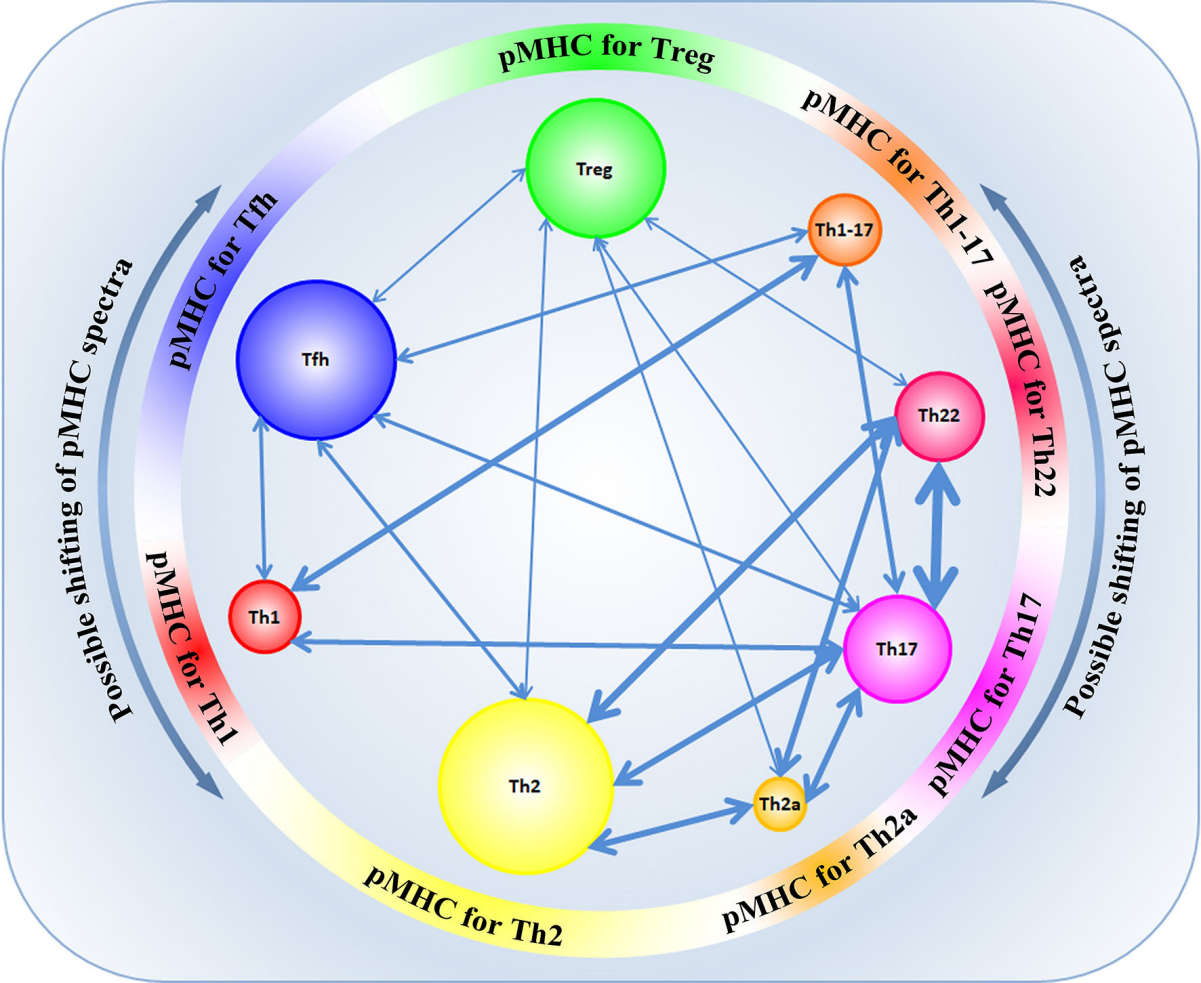
The landscape of presentation shapes different CD4^+^ subpopulations according to their CDR3 physicochemical properties and specificity. Possible shifts in pMHC spectra can contribute to transdifferentiation between some subpopulations of CD4^+^ T cells. The width of the arrows reflects the number of common TCR clonotypes between subpopulations.

Transdifferentiation between the different T-cell subpopulations is well-known. However, not long ago, it was established that functionally different subpopulations of CD4^+^ cells expressed TCRs with different physicochemical properties and had different profiles of VDJ recombination, which affected their tendency to differentiate into each other (48). In their study, Kasatskaya et al. (48) focused on some characteristics of the CDR3 region in different subpopulations of T cells. The authors of that study evaluated different properties of amino acids in the CDR3 loop, the hydrophobicity of the loop (Kidera factor 4) (49), the length of the CDR3 loop, the predicted averaged binding energy of the TCR-pMHC (50, 51), and some other parameters that generally influence the affinity of Ag-specific TCR-pMHC interaction and the degree of TCR cross-reactivity (48). The study of these parameters revealed the differences in the physicochemical properties of the CDR3 TCR loop at the level of different subpopulations of T cells. It was shown that Treg cells have TCRs with high cross-reactivity, while follicular helpers Tfh have TCRs with minimal cross-reactivity (48). TCRs of Treg cells exert relatively higher affinity to self-antigens, bind cognate pMHC ligands less specifically and have lower averaged energy of TCR-pMHC binding than Tfh cells that bind cognate pMHC ligands with high affinity and have a higher energy of TCR-pMHC binding, which agrees with previous data (52–54). At the same time, such differences were also observed in other subpopulations: amino acidic characteristics of the CDR3 loop among the populations Th1/Th1-17/Th17 were similar to the characteristics of Tfh, while among populations Th22/Th2a/Th2, there was a similarity with Treg cells (48). Besides, different subpopulations of T cells were distinguished by a diversity of TCR repertoires. The highest diversity was observed in the subpopulation Tfh. A relatively high TCR diversity was observed in the subpopulations Th2, Th17, Th1, and Treg, while subpopulations Th22 and Th2a had signs of oligoclonal expansion, which indicated Ag-specific proliferation in these subpopulations (48). The physicochemical properties of TCRs are different in naïve TCR T cells and memory T cells, which was shown for populations of CD4^+^ and CD8^+^ lymphocytes. At the same time, for naïve nTreg and memory mTreg cells, such differences were not revealed (48, 55). Thus, the functional specialization of T cells depends on the structure of the CDR3 region and could be determined by the interaction of TCRs with the respective diversity of pMHC epitopes for each T-cell population (**Figure 3**). A paired analysis of the overlap of CDR3*β* diversity of different subpopulations of T cells revealed certain consistency in the transdifferentiation in healthy donors. A high plasticity was revealed between the functionally close populations Th17/Th22, Th17/Th2, Th22/Th2, Th2/Th2a. Lower plasticity was revealed between other populations Th17/Treg and Th1/Th17. At the same time, Tfh and Treg subpopulations were characterized by a high discreteness and had few CDR3*β* sequences common with other subpopulations of T cells (**Figure 3**) (48). Such data were first obtained for healthy donors and agreed with the data obtained *in vitro* or on animal models (38, 56, 57). Taking into account that the functional specialization of the subpopulation of T cells could be defined by specific interaction between TCR-pMHC and physicochemical properties of the CDR3 loop, the changes in the presentation landscape of antigens can lead to changes in the clonal structure of certain subpopulations of T cells. In some cases, such transitions of Ag-specific clones between subpopulations of T cells are involved in pathological processes. For example, an important role of a transition Treg ↔ Th17 was established in patients with different autoimmune conditions, graft rejection, and oncologic processes (58–63). Besides, plasticity Th17 → Th1 was revealed in patients with juvenile idiopathic arthritis and Crohn’s disease. In patients with allergic conditions and bronchial asthma, an enhanced transdifferentiation Th17 → Th2 was observed (64, 65). An imbalance between the subpopulations Th1/Th2 explains the pathogenesis of allergic and some oncologic diseases. However, in this case, an incorrect functional specialization of certain Ag-specific clones in the ontogenesis resulting from the changes in the conditions of presentation of the respective antigens is observed rather than a transdifferentiation (66–68).

Similar to CD4^+^ lymphocytes, an imbalance between effector CD8^+^ Teff and regulatory CD8^+^ Treg cells is significant for the maintenance of the immune equilibrium. Along with humoral factors of suppression, CD8^+^ Treg cells can exert Ag-specific suppressive activity mediated by the interaction with antigen-representing cells (69). Shifts in the represented antigen spectra and changes of conditions of their presentation can contribute to the irrelevant CD8^+^ Teff ↔ CD8^+^ Treg transdifferentiation (70). It was shown that such plasticity between subpopulations of CD8^+^ lymphocytes significantly contributed to the pathogenesis of different autoimmune and infectious diseases and oncological processes and took part in the graft rejections (69, 71–73). Still, despite the present achievements in the understanding of the organization of the T-cell repertoire, the identification of certain clones involved in the pathogenesis of different diseases attracts the attention of scientists in modern immunology and opens perspectives for personalized medicine.

## Organization of the Human B-Cell Repertoire

**Figure 4:**
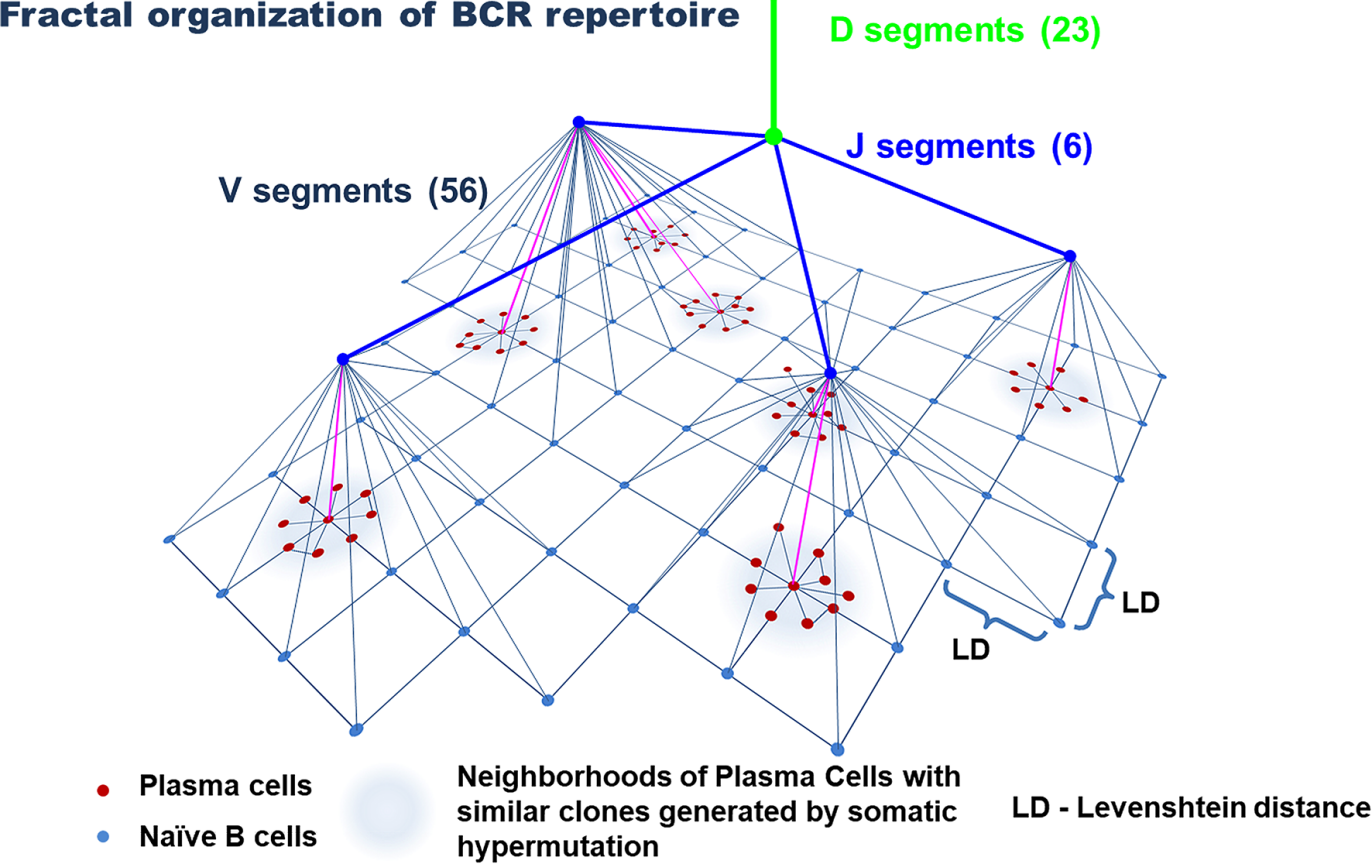
Model of the organization of the B-cell repertoire. Tree-like structures generated by VDJ recombination and nucleotide additions/deletions and a star-like structure for plasma cells likely generated by somatic hypermutation. The uniform distribution of naive B cells in the similarity layer schematically reflects a homogeneously interconnected network (by Levenshtein distances) of these cells, in contrast to plasma cells, that form highly interconnected subnetworks of similar clones. The number of V, D, and J segments is indicated for the IgH chain.

The immunoglobulin gene rearrangement of B cell in the bone marrow results in the formation of a highly diverse repertoire of naïve (antigen-inexperienced) B cells that get into the peripheral circulation (74, 75). Similar to T cells, this process occurs under the influence of a complex of RAG, TdT, and a number of enzymes. Their activation induces V(D)J recombination and P- and N-insertions in the CDR3 loop of naive B cells (75–78). Further diversification of the BCR repertoire is associated with SHMs that occur in a cell under the influence of activation-induced deaminase (AID) in the peripheral lymph nodes with a cognate antigen (79, 80). This process underlies the affinity maturation of antibodies and targets an increase in the specificity of antigen recognition (75). Similar to T cells, the frequency distribution of clones in the general repertoire of B cells complies with power-law distribution (**Figure 1**) and agrees with the general biological type of Pareto distribution (81, 82). In other words, the BCR repertoire contains a relatively low number of dominant clones and an extremely high diversity of minor clones that form a long tail of distribution. Besides, as for T cells, the repertoires of naïve B cells are characterized by a tree-like fractal organization (**Figure 4**) (82, 83).

However, the architecture of the BCR repertoire has some peculiarities that are closely associated with the process of SHM and the formation of memory plasma cells. The diversity of the repertoire of these cells is significantly lower than the diversity of naïve B cells (84), which is associated with the history of antigen challenges that an organism faces throughout its life. At the same time, a tree-like structure of the repertoire of naïve B cells, which is generated due to VDJ recombination and nucleotide insertions, is replaced by a star-like structure for memory B cells and plasma cells, which is associated with the process of SHM (**Figure 4**) (82, 85). Such star-like structures reflect the process of activation of one or several B-cell clones closed by specificity. In the course of further expansion and SHMs, these B-cell clones form a set of antigen-experienced B-cell clone neighboring in the common space of CDR3 sequences. Part of these cells later becomes plasma cells. In this case, for the evaluation of similarity/closeness of the clones, Levenshtein distances were used (82, 86). Finally, the activity of AID is capable of mediating the shift of heavy chains from IgM/IgD to IgG, IgA, or IgE during SHM (74). It should be noted that despite the high personalization of BCR repertoires, different individuals have a similar organization of BCR and antigen repertoires (82, 87), which indicates general principles of the formation of BCRs and Ig diversity in different individuals. At the same time, the diversity of naïve B cells is affected by self-antigens and the repertoire of naïve B cells is limited by positive and negative selection (35, 36). The diversity of plasma cells directly depends on the diversity of antigen challenges within the life and depends on the functional activity of T cells, which is confirmed by a significant decrease in SHM in T cell-deficient mice (36, 87).

It is well-known that B cells play a central role in humoral immunity as antibody producers, can express some cytokines, and act as antigen-presenting cells (88–90). During the past years, many studies have been dedicated to the subpopulation of B cells with regulatory functions that were called B regulatory cells (Bregs) (91–94). Bregs exert their functions due to the production of anti-inflammatory cytokines, inhibit different populations of immune cells, and can induce the formation of Tregs from effector T cells acting as tolerogenic antigen-presenting cells, which do not exclude the Ag-specific effect of Bregs (91, 95, 96). Similar to T cells, irrelevant induction of Bregs and an imbalance between effector and regulatory B cells play a significant role in the pathogenesis of different autoimmune and oncologic processes, in patients with chronic infections and graft rejections (97–101). However, in this case, a transition Beff ↔ Breg could be primarily associated with the peculiarities of the microenvironment and only indirectly mediated by the shifts of spectra of the presented antigens and the conditions of their presentations *via* T cells (91, 102–104). Still, in some cases, the induction and functional activity of Bregs depend on the recognition of cognate antigens by Breg cells; and the suppression activity of Bregs can be mediated by direct B–T cellular interaction, which confirms the possibility of the Ag-specific effect of Bregs (105, 106). Thus, the conditions of the microenvironment and spectra of B-dependent antigens in the microenvironment of B cells influence their functional specialization; and an irrelevant transdifferentiation Beff ↔ Breg can underlie the pathogenesis of different pathologies.

An extremely high diversity of Ag-recognizing receptors of T and B cells provides the formation of qualitatively new properties that distinguish adaptive immunity from innate. The most important of them is the specificity of antigen recognition. Another important property is universality, *i.e.* the adaptive immune system can potentially specifically recognize any antigen of all possibilities. Redundancy—the same antigen can be recognized by different Ag-recognizing receptors due to cross-reactivity and the fact that one antigen can have different epitopes. Clones that are close by their specificity can duplicate and replace each other during the formation of the immune response. This underlies the robustness of adaptive immunity. In general, these properties provide the reliability of the immune system functioning and reflect the qualitative–quantitative transition, when a high diversity of Ag-recognizing receptors provides a qualitatively new level of the immune system functioning. However, changes in the conditions of antigen presentation can lead to situations when a disturbance of functional specialization of some subpopulations of T and B cells occurs, which can underlie the pathogenesis of different pathologies and be the main factor in the disturbances of the adaptive immunity.

## Presentation Landscape

Rearrangement of a genome underlies a colossal diversity of Ag-recognizing receptors (17, 75, 107). However, the final formation of naïve repertoires of T and B cells is observed during the process of positive, and then, negative selection and depends on the diversity of the antigens presented in the thymus (108, 109) and the diversity of self-antigens in bone marrow (110, 111). In the past years, more data have been accumulated that confirm the influence of allele variants of MHC on the formation of individual TCR landscape (25–27, 112, 113).

## T-Cell Presentation Landscape

The significance of MHC restriction for the development of T cells is well-known and can be illustrated by a recent study. It showed that during positive and negative selection, a selection of T cells with certain properties of the CDR3 loop occurred (114). Thus, during the process of positive selection, MHC restriction provides the selection of TCRs with the length of CDR3 (8–13 amino acid residues) and limits the selection of TCRs with positively charged and hydrophobic amine acid residues in the CDR3 loop. During the process of negative selection, it prevents the selection of TCRs with the residues of cysteine in the Ag-binding regions of the CDR3 loop (114). Probably, in this case, the selection of TCRs is primarily influenced by the physicochemical properties of MHC molecules and not certain epitopes in their composition. It should be noted that MHC restriction does not lead to the selection of TCRs with certain sequences of amino acids in the CDR3 loop and preserves high randomness of amino acid sequences in the CDR3 loop and sufficient diversity of TCRs for the recognition of the variety of potential antigens (9, 114, 115).

Apart from the common physicochemical properties of MHC molecules, an important role in the formation of the naïve repertoire of TCRs is played by epitopes presented in the thymus as a part of MHC molecules. The formation of the central auto-tolerance occurs due to the independent activity of transcriptional factors AIRE and Fezf2 that induce the expression of different tissue-restricted antigens by medullary thymic epithelial cells (mTECs) and thymic B cells (AIRE), which provide the elimination of self-reactive T cells during negative selection (109, 116–119). At the same time, some T cells, that exert a relatively high affinity to self-antigens, become Treg cells (38, 117). The affinity of TCR Treg cells to self-antigens is 100-fold lower than in the self-reactive T cells that undergo negative selection (38, 120). It was established that the transcriptional factors AIRE and Fezf2 provided the expression of nearly 60% of tissue-restricted antigens presented in the thymus (116). It is suggested that other antigens are presented in the thymus by different subpopulations of dendritic cells (DCs) (121). Presently, at least three DC subpopulations are known to provide the presentation of antigens in the thymus: CD8α^+^, Sirpα^+^, and B220^+^ plasmacytoid dendritic cell (pDCs). CD8α^+^ DCs occupy ~50% of the pool of thymic DCs (121), develop from the precursors in the thymus (122), and present tissue-restricted antigens, obtained from mTECs during trogocytosis or uptake of extracellular matrix, to T cells (123). Sirpα^+^ DCs occupy ~20% of the pool of thymic DCs, are found primarily in the corticomedullary perivascular spaces (124), and present antigens taken up from the bloodstream or acquired in the peripheral tissues before the migration to the thymus (121). The remaining ~30% of the pool of thymic DCs represent B220^+^ pDCs and present antigens obtained primarily on the periphery before the migration to the thymus (121). The presentation of self-antigens by the thymic DCs provides a negative selection of self-reactive CD4^+^ and CD8^+^ cells and probably contributes to the formation of Treg cells. However, precise mechanisms of this process are understudied (121, 125, 126). It is suggested that the migration of DCs from the periphery and the pathologic migration of B cells to the thymus (127) create the risks of inadequate formation of the central tolerance, for example, to the tumor or infectious antigens. However, this assumption requires experimental confirmation. Besides, it should be noted that the mechanisms of central tolerance do not exert absolute efficiency, and some self-reactive T cells can get to the peripheral bloodstream, which should be suppressed by Treg cells in normal conditions (54). This creates additional risks for the development of autoimmune diseases.

Thus, as a result of the described process, two varieties of T cells are formed that leave the thymus: T cells with relatively high affinity to self-antigens (Tregs) and T cells with low affinity to self-antigens (future anti-pathogen T cells) (54). After leaving the thymus, recent thymic emigrants (RTEs) migrate to the peripheral lymph nodes. During the maturation under the influence of the microenvironmental factors, they form a variety of mature naïve T cells (128). It is suggested that on the periphery, RTEs are subject to additional selection that is provided by MHC molecules in the peripheral lymph nodes. Only some RTEs with certain physicochemical properties of the CDR3 loop become mature naïve T cells (128). Probably, this stage of peripheral selection is necessary for the culling of potentially self-reactive T cells that manage to avoid negative selection in the thymus and deletion of T cells that cannot recognize effectively MHC molecules (48, 55, 128). At the same time, it is suggested that Treg cells do not undergo this stage of additional peripheral selection, which is confirmed by the lack of differences in physicochemical properties of the CDR3 loop between immature and mature subpopulations of Treg cells (48). Thus, the presentation landscape on the periphery provides an additional stage of the selection of CD4^+^ and CD8^+^ cells. As a result, a formation of the repertoire of mature naïve T cells occurs that will further go through a functional specialization according to their physicochemical properties and the specificity of their TCRs and form the main subpopulations of Th lymphocytes (48, 55, 129–131). It is evident that the changes in the landscape of presentation of antigens associated with different antigen challenges in the ontogenesis will influence the further formation of the TCR repertoire and clonal organization of different subpopulations of T cells in a certain individual. At the same time, it should be noted that the personalization of TCR repertoires of CD4^+^ and CD8^+^ is significantly provided by the individual set of allele variants of MHC genes (26, 31–33, 112).

## Genetic Variants of MHC Molecules Influence the Landscape of Recognition

During the past decade, a lot of attention has been paid to the study of the influence of MHC allele variants on the repertoire of TCRs in different individuals. Associations were revealed between MHC genetic variability and the profiles of expression of TCR V genes (132). It was established that such associations were provided not only by the contact of TCRs with a peptide in the MHC complex but also by a physical contact between V-regions of the TCR *β*-chain and complementary regions of MHC molecules (132, 133), which indicated a direct influence of MHC genotypes on the formation of individual TCR repertoires. This agrees with the assumption of Niels Jerne on the co-evolution of MHC and TCR genes for a better predisposition to interact with each other (134). Still, the influence of the MHC genotype is primarily observed on the CD8^+^ lymphocytes. This is explained by a closer contact between TCRs of CD8^+^ cells and MHC-I molecules, while the regions CDR1 and CDR2 in TCRs of CD4^+^ cells have a weaker contact with complementary regions of the MHC-II molecules, and the region CDR3 primarily contacts with a peptide in the MHC-II complex (133, 135). This could provide the lack of influence of individual polymorphism of MHC-II genes on the diversity of the CD4^+^ repertoire, while a higher polymorphism of the MHC-I gene in heterozygotes is associated with a higher diversity of CD8^+^ cells (27). Still, a recent study on animal models showed a direct influence of MHC-II allele variants on the diversity and clonal organization of the CD4^+^ repertoire, including Treg cells (112). This confirms the hypothesis that allele variants of MHC-I and MHC-II play an important role in the formation of TCR repertoires of CD4^+^ and CD8^+^, respectively. Besides, it was shown that mutations in the conservative regions of MHC-I and MHC-II that contact with the complementary regions of TCRs influence the profiles of expression of TRAV and TRBV in the CD4^+^ and CD8^+^ repertoires and change their clonal organization (136, 137).

Thus, it is evident that the individual HLA phenotype defines epitope spectra that could be presented with the highest possibility, *i.e.* HLA phenotype is responsible for the formation of immunopeptidomes of MHC-I and MHC-II-associated antigens (138, 139). In turn, this affects the selection of T cells in the thymus, plays an important role in the formation of individual TCR repertoires on the periphery, and determines the individualization of the immune response. Such association between HLA genes and TCR repertoires reflects a close functional and phylogenetic association between innate and adaptive immunity.

## Potential Risks of Immune Disequilibrium

The equilibrium in the immune system is achieved due to fine coordination between the innate and adaptive branches of immunity. Potential risks of the immune disequilibrium can be associated with different genetic factors, all possible antigenic challenges, and the influence of unfavorable factors of the environment.

It is well-known that there is a genetic predisposition to auto-immune diseases (ADs). It is hypothesized that the influence of different HLA variants and other genes associated with ADs in combination with epigenetic factors and unfavorable exogenous conditions contributes to the development of ADs (140). However, the presence of genetic predisposition does not always lead to the realization of the risk of ADs. Probably, an additional trigger is required (long-term lymphopenia or some immune regulatory disturbance) (141, 142). During the past two decades, the role of homeostatic proliferation in the development of ADs has been widely discussed (54, 143–147). This is a physiological process of the quantitative restoration of the peripheral pool of T cells after lymphopenia of any etiology by means of the antigen-specific proliferation of lymphocytes under the influence of IL-7 and IL-15 that could acquire pathological traits depending on the depth of lymphopenia (54, 148, 149). It was shown that this process could result in the selection of potentially self-reactive clones of T cells due to the competition for the contact of TCRs with self-pMHC, in a decrease in the diversity of the general TCR repertoire, and in a decrease of functional activity of Treg cells because of the deficiency of IL-2 in the conditions of lymphopenia (54, 150–153). Besides, a disturbance of the functional specialization of Treg cells and their conversion into pathogenic Th lymphocytes can occur (154). Some studies showed that Treg cells could not suppress the proliferation of T cells that received a strong TCR signal under the influence of IL-7 and IL-15, which was important in the context of homeostatic proliferation when a strong TCR signal gives advantages to T cells in the competition for the factors of survival (155, 156). This fact is interesting taking into account that AD-associated variants of MHCs contribute to a better presentation of antigens associated with the disease, and thus, homeostatic proliferation can contribute to the expansion of self-reactive T-clones in people with genetic predisposition (157, 158). Thus, a shift of spectra of the presented antigens towards self-antigens that are provided by AD-associated HLA variants and mediated *via* homeostatic proliferation of the changes in the clonal organization of TCR repertoires can underlie the disturbances in the immune equilibrium in patients with ADs.

Homeostatic proliferation can lead to a favorable antitumor immune response (159, 160). This response is formed as a result of polyclonal homeostatic expansion in the lymph nodes and is characterized by CD8^+^-cell cytotoxicity, an increase in the concentration of IFN*γ*, and the formation of memory cells (159). Besides, some data indicate that the shift in focus of homeostatic proliferation from CD8^+^ to CD4^+^ cells can be one of the causes of the development of ADs (159, 161). At the same time, the homeostatic proliferation of B cells does not lead to negative effects because it is exerted *via* an Ag-independent pathway and does not influence the diversity and clonal organization of the BCR repertoire (162, 163).

Probably, homeostatic proliferation can also contribute to a decrease in the general diversity of TCRs and the TCR diversity of naïve T cells with age (23), which negatively affects the protective function of the immune system against infections or other antigenic challenges in senior age (23, 164). It was shown that increased sensitivity to viral and oncological disease was associated with a decrease in the diversity of TCRs and connected with the formation of holes in the TCR repertoires (164–167).

The conditions of the microenvironment can significantly affect the functional activity of T and B cells causing their activation or leading to anergy and inducing the formation of Treg and Breg cells. An inflammatory microenvironment and co-stimulating signals that are transmitted during contact with the neighboring cells can lead to non-specific activation of different lymphocyte clones due to a so-called bystander effect (168). Since self-antigens can be present in the site of inflammation or immune response to an infection and any other antigen, the bystander effect can potentially cause unfavorable activation of self-reactive clones and increase the risk of ADs (168–171). Probably, a functional modulation of the TCR activation threshold due to the factors of co-stimulation and inflammatory microenvironment can contribute to the non-specific activation of T cells (172, 173). It was established that as a result of the bystander effect, a disturbance in the functional specialization of different subpopulations of lymphocytes could occur, for example, a transition of Treg cells into pathogenic Th lymphocytes, which could be also associated with the risk of the development of ADs (174). In other words, the bystander effect can lead to irrelevant Ag-independent activation of self-reactive lymphocytes and their expansion changing the structural organization of TCR and BCR repertoires at the level of separate clones and can contribute to the development of ADs.

Apart from the activation of lymphocytes, the factors of the microenvironment can cause anergy or even induce lymphocytes with regulatory functions by the Ag-independent bystander suppression. Similar effects are observed in the microenvironment of tumors that express some suppressor factors forming a tolerogenic medium and avoiding the immune surveillance (175, 176). At the same time, in the microenvironment of the tumor, a population of tolerogenic dendrite cells is formed. These cells are responsible for the formation of tumor-specific tolerance that is provided by T and B lymphocytes with regulatory functions (177–180). Besides, there is a possibility of the formation of central tolerance due to a migration of some dendrite cells loaded with tumor antigens to the thymus, wherein they can potentially be involved in the process of negative selection of T cells (121, 124–126). However, this assumption is hypothetical, and this issue requires additional research. Thus, the changes in the conditions of presentation of tumor antigens can shift the immune response from the immunogenic to tolerogenic and result in the respective changes in the clonal organization of T- and B-cell repertoires (181, 182). Besides, it changes the functional specialization of different lymphocyte populations providing the progression of the tumor growth.

## Conclusion

The maintenance of the equilibrium in the immune system is an intricate dynamic process associated with constant changes in the landscapes of presentation and recognition, wherein genetic HLA variants play an important role, influence the formation of TCR repertoires, and determine the individualization of the immune response. In general, the disturbance of the immune equilibrium (autoimmune, infectious, or oncogenic process) is associated with the changes in the conditions of presentation and the spectra of the presented antigens, as well as with the transformation of T- and B-cell repertoires and a shift in the functional specialization of some T and B cells. In this case, the most important role is played by the genetic background and the influence of external environmental factors.

It is suggested that the study of genetic HLA variants and immunopeptidomes associated with a disease in a certain individual and the identification of certain clones of T and B cells involved in the pathogenesis of the disease will allow using personalized approaches to the therapy of different pathologies based on a targeted, specific effect on certain pathology mechanisms.

## Author Contributions

DS contributed to the conception, drafting of the manuscript, and design. VT contributed to the conception and revision. VK contributed to the revision and final approval of the manuscript. All authors contributed to the article and approved the submitted version.

## Funding

The research was supported by the grant of the Russian Science Foundation project no. 21-65-00004, https://rscf.ru/project/21-65-00004/.

## Conflict of Interest

The authors declare that the research was conducted in the absence of any commercial or financial relationships that could be construed as a potential conflict of interest.

## Publisher’s Note

All claims expressed in this article are solely those of the authors and do not necessarily represent those of their affiliated organizations, or those of the publisher, the editors and the reviewers. Any product that may be evaluated in this article, or claim that may be made by its manufacturer, is not guaranteed or endorsed by the publisher.
